# Preservation of laryngeal function improves outcomes of patients with hypopharyngeal carcinoma

**DOI:** 10.1007/s00405-014-3115-2

**Published:** 2014-06-10

**Authors:** Tong Jin, Xuezhong Li, Dapeng Lei, Dayu Liu, Qiuan Yang, Guojun Li, Xinliang Pan

**Affiliations:** 1 Department of Otorhinolaryngology, Qilu Hospital, Shandong University, Key Laboratory of Otolaryngology, Ministry of Health, 107 West Wenhua Road, Jinan, Shandong 250012 People’s Republic of China; 2 Department of Radiation, Oncology Center, Qilu Hospital of Shandong University, Jinan, Shandong 250012 People’s Republic of China; 3 Department of Head and Neck Surgery, The University of Texas MD Anderson Cancer Center, 1515 Holcombe Boulevard, Unit 1445, Houston, TX 77030-4009 USA

**Keywords:** Function preservation, Hypopharyngeal squamous cell carcinoma, Laryngectomy, Pharyngectomy, Survival, Life quality

## Abstract

This study compares clinical characteristics and survival between patients with and without laryngeal function (LF) preservation during surgical treatment for hypopharyngeal carcinoma. We retrospectively reviewed 485 cases of hypopharyngeal carcinoma treated at a single institution for analysis. There were 337 cases with and 148 cases without LF preservation after surgery. Preservation of LF was complete in 237 patients and partial in 100 patients. There were significant statistical differences between the preservation group and the group without preservation in T-stage (*P* < 0.001), overall staging (*P* < 0.001), and tumor sites (*P* < 0.001) except the N-stage (*P* = 0.240). The patients with LF preservation had significantly better overall survival (log-rank, *P* = 0.005) and a lower risk of death than those without LF preservation (HR 0.62, 95 % CI 0.43–0.97), after multivariable adjustment. Treatment with surgery in combination with radiotherapy is still the favorable choice for patients with hypopharyngeal carcinoma. The maximal restoration of pharyngoesophageal continuity and function improves survival for patients whose tumors are excised completely for the preservation of LF and laryngeal and pharyngeal reconstruction.

**Electronic supplementary material:**

The online version of this article (doi:10.1007/s00405-014-3115-2) contains supplementary material, which is available to authorized users.

## Introduction

Hypopharyngeal carcinoma occurs most commonly in the pyriform sinus, followed by the posterior wall of the hypopharynx; it occurs less commonly in the postcricoid region. Hypopharyngeal carcinoma has the worst prognosis of all neoplasms of the upper respiratory-gastrointestinal tract, with a 5-year survival rate of 30–50 % [1]. This poor prognosis is due to the disease being relatively asymptomatic in the early stages, to its tendency for submucosal spread, and to the early occurrence of lymphatic/distant metastasis [2]. Symptoms develop as a consequence of encroachment on the larynx or obstruction of the alimentary tract. Cervical lymph node metastasis is a common presenting symptom and denotes an advanced stage of disease with poor prognosis.

The treatment of hypopharyngeal carcinoma usually includes surgery that involves the larynx, which is essential for speech, respiration, and deglutition. Because laryngeal integrity is usually maintained in the treatment of early stage hypopharyngeal carcinoma, the preservation of laryngeal function (LF) is relatively easy. However, in advanced hypopharyngeal carcinoma, preservation of LF is difficult because the deeper the progression of the tumor into the larynx, the more likely the surgical treatment will include resection of the full thickness of the larynx. Preservation of LF has become a major area of concern and study, and progress has been made in recent years [3–8]. In this study, we retrospectively reviewed the clinical files of patients with hypopharyngeal carcinoma who were treated at our institution from January 1999 to December 2009 and evaluated the clinical characteristics and compared the survival of patients with or without preservation of LF after surgical treatment.

## Methods

### Patients and data

From January 1999 to December 2009, 485 patients with hypopharyngeal carcinoma had been operated in the Department of Otolaryngology, Qilu Hospital of Shandong University, Jinan, China. All patients had a definite pathological diagnosis of squamous cell carcinoma of the hypopharynx before surgery. Primary tumor subsite, clinical stage, treatment, and vital status were recorded from the medical records as assessed between the initial and final patient contact recorded. All patients completed an Institutional Review Board-approved informed consent form before enrollment.

### Surgical management

Routine presurgical examinations included X-ray barium meal examination, enhanced cervical computed tomography, laryngoscopy, and biopsy. The primary lesions of all 485 patients were resected. The approach varied depending on the location of the lesion, but all aimed to fully expose the tumor to the naked eye. Parts of the ipsilateral thyroid cartilage, the ipsilateral hyoid bone, and the cricoid cartilage were resected, if necessary, to expose the carcinoma. The thyroid cartilage cornu superius approach was chosen for primary lesions located in the pyriform sinus. One-third to one-half of the posterior part of the ipsilateral thyroid cartilage and the ipsilateral hyoid bone was resected to expose the tumor. The epiglottic vallecular cervical esophageal approach was chosen when the patients had advanced pyriform sinus carcinoma. The thyroid cartilage cornu superius and hyoid bone approaches were used to resect primary lesions located in the posterior wall of the hypopharynx. The thyrohyoid membrane and pyriform sinus approaches were chosen to resect postcricoid area carcinoma. A tumor margin of at least 1.5 cm was considered optimal. If the esophagus was invaded, the esophageal incisal edge was greater than 3 cm. Among the patients whose laryngeal function was preserved, epiglottal tissue was used most commonly to reconstruct the larynx. All patients underwent neck dissection, no matter whether the cervical lymph nodes metastasis diagnosis before surgery exited or not. If the primary carcinoma invaded the midline of pharynx or a clear diagnosis of contralateral lymph node metastasis was confirmed by physical examination or imaginably before surgery, a bilateral neck dissection was chosen. Among the 630 total neck dissections, only 50 were comprehensive; the others were selective neck dissections. The surgical procedures or methods are illustrated in supplemental Figure 1.

### Adjuvant radiotherapy

All 485 patients received adjuvant radiotherapy for 20 days to 2 months after surgery. The dosage of the postoperative radiation was 50–60 Gy in 400 cases and 75 Gy in the other 85 cases who had advanced cancer or extra-capsular invasion of lymph nodes.

### Follow-up and statistical analysis

We used χ^2^ tests to examine differences between the patients with and without LF preservation in the distributions of clinical characteristics including T-stage, N-stage, and tumor sites. The primary endpoint in this study was overall death. Overall survival (OS) was defined as the time from first diagnosis to death from any cause or last follow-up. Participants who were alive at the end of the study period or lost to follow-up were considered censored. Medical record review to determine the follow-up status of all patients was performed under the direct supervision of the staff head and neck surgeon. Survival rates were calculated using the Kaplan–Meier method, and the differences between the survival curves were examined using the log-rank test. Univariate Cox proportional hazards regressions were applied to estimate the individual hazard ratio (HR) for the overall survival. The significant variables in the univariate analyses (*P* < 0.05) were then put into the multivariate analysis. The HR with 95 % confidence interval was measured to estimate the hazard risk of individual factors. SPSS software, version 19.0 (SPSS, Inc., Chicago, IL, USA), was used for the statistical analyses. All *P* values were two-sided, and statistical significance was defined as *P* < 0.05.

## Results

We identified 485 patients with hypopharyngeal carcinoma, including 445 males and 40 females ranging in age from 26 to 82 years (mean age 58.5 years). A total of 374 patients had pyriform sinus carcinoma, 87 had posterior wall carcinoma, and 24 had postcricoid area carcinoma. The patients’ TNM classifications are shown in Table 1. None of the patients had distant metastasis (all *M* = 0).

**Table 1:** Clinical characteristics of patients with and without laryngeal function preservation (*N* = 485)

Clinical characteristics	Total		LF preserved		LF not preserved		*P* value*
	*N*	%	*N*	%	*N*	%	
Total patients	485	100	337	69.5	148	30.5	
T stage							<0.001
T1–2	150	30.9	143	42.4	7	4.7	
T3–4	335	69.1	194	57.6	141	95.3	
N stage							0.240
N0–1	320	66.0	228	67.7	92	62.2	
N2–3	165	34.0	109	32.3	56	37.8	
Overall staging							<0.001
I–II	54	11.1	53	15.7	1	0.7	
III–IV	431	88.9	284	84.3	147	99.3	
Tumor site							<0.001
Pyriform sinus	374	77.1	284	84.3	90	60.8	
Postcricoid area	24	5.0	7	2.0	17	11.5	
Posterior wall	87	17.9	46	13.6	41	27.7	

*LF* laryngeal function

* P values were calculated from Chi square test

We preserved LF in 337 of the 485 cases for a LF preservation rate of 69.5 %. Table 1 shows the distribution of T stage, N stage, overall clinical stage, and primary tumor sites between patients with and without LF preservation, and the distribution was significantly different between the two groups except for N stage. Among the 337 cases with LF preservation, 94 cases did not need LF reconstruction because the laryngeal integrity was maintained; the other 243 cases were reconstructed using the epiglottis (147 cases), sternohyoid myofascial flap (41 cases), thyroid perichondrium (33 cases), or platysma myocutaneous flap.

**Fig. 1:**
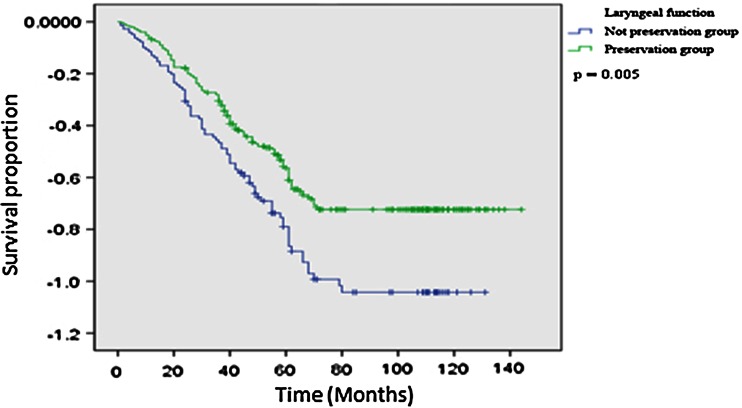
Kaplan–Meier estimates for the survival of patients according to LF preservation

The response rate of follow-up was 96.3 % (467/485). Among 467 cases with information of follow-up, 252 cases died, 237 cases were still alive, and 22 cases were lost to follow-up (11 patients moved to other locations, 8 cases did not voluntarily respond to our research interviewers, and 3 with unknown reasons). The overall survival rate was 48.0 %. The survival rates of groups with and without LF preservation were 52.2 and 38.5 %, respectively (*P* = 0.005) (Fig. 1). Multivariable Cox proportional hazards regression analysis was performed to evaluate the association between LF preservation and overall risk of death in patients with hypopharyngeal carcinoma. The adjusted confounders included age, sex, T stage, N stage, overall stage, tumor site, and treatment. As shown in Table 2, compared with the patients without LF preservation, patients with LF preservation had a significantly reduced risk of overall death (HR 0.62, 95 % CI 0.43–0.97).

**Table 2:** Multivariable analysis of the association between LF preservation and risk of death in patients with and without laryngeal function preservation (*N* = 485)

Laryngeal function	Total		Death		HR^a^, 95 % CI
	*N*	%	*N*	%	
Total patients	485	100	252	52.0	
LF not preserved^b^	148	30.5	91	61.5	1.00
LF preserved	337	69.5	161	47.8	0.62 (0.43–0.97)

*HR* hazard ratio

^a^Adjusted for age, sex, T, N, overall stage, tumor sites, and treatment

^b^Reference group

**Fig. 2:**
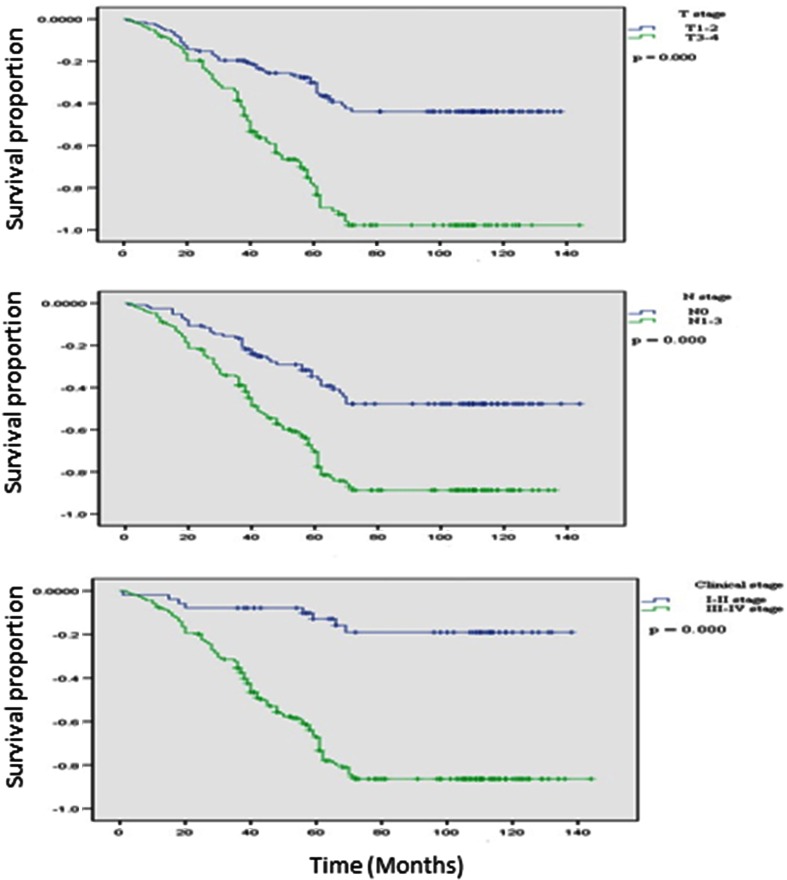
Kaplan–Meier estimates for survival according to T, N, and overall stage among patients with LF preservation (*N* = 337)

**Fig. 3:**
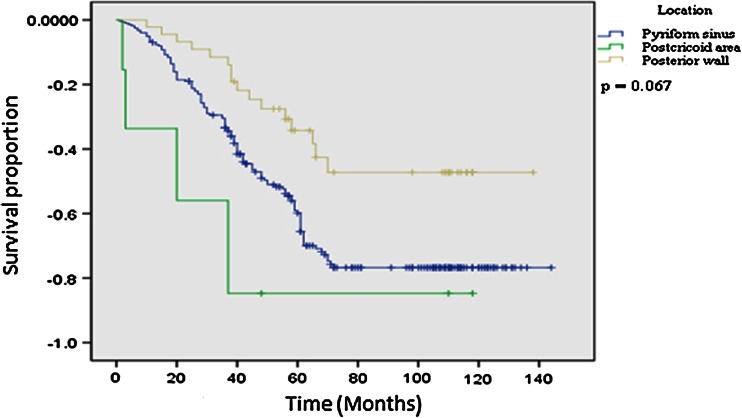
Kaplan–Meier estimates for survival according to tumor site among patients with LF preservation (*N* = 337)

Because we found that LF preservation may affect the prognosis and quality of life of patients with hypopharyngeal carcinoma and that the patients with LF preservation had a significantly higher survival rate and a lower risk of overall death than those without LF preservation, we further explored the associations only among patients with LF preservation. As we expected, the patients with early T, N, or overall disease stage had a significantly better survival rate (Fig. 2) and a lower risk of overall death (data not shown) than those with advanced disease stages. Furthermore, the patients with hypopharyngeal carcinoma located in the posterior wall had a higher survival rate than those with pyriform sinus and postcricoid area carcinomas, although the difference did not reach significance (Fig. 3). Similar analyses were not performed for patients without LF preservation because of the relatively small numbers of cases and events. However, such analyses will be performed when we have a large enough number of these patients. Finally, among the 337 cases with LF preservation, 237 cases (70.3 %) had overall LF recovery (respiration, speech and deglutition); 100 cases (29.7 %) had partial LF recovery (speech and deglutition only). Among the 148 patients without LF preservation, 141 (95.3 %) had recovery of deglutition.

## Discussion

The prognosis of hypopharyngeal carcinoma is still poor because of poor tumor differentiation, local infiltration, and metastasis [9]. In addition, because it is usually asymptomatic in the early stages, most hypopharyngeal carcinomas are in an advanced stage when clinically diagnosed [2].

To improve the survival and quality of life of patients with hypopharyngeal carcinoma, LF can be preserved during surgery without reducing the local control rate. Before the decision to attempt LF preservation is made, several key surgical processes should be taken into consideration as discussed below. The choice of surgical approach depends on the location of the hypopharyngeal carcinoma, which affects its development and biological behavior. For example, carcinomas arising from the lateral wall of the pyriform sinus always invade the posterior part of the thyroid cartilage, pass through the posterior edge of the cartilage to the outside, and involve the tissue of the larynx or thyroid gland. During the resection of the tumor at the lateral wall, the affected board of ipsilateral thyroid cartilage has to be split directly in a longitudinal direction, and then the cavity of the hypopharynx is opened. The remnant board of thyroid cartilage can be pulled forward to achieve a more spacious surgical approach. Meanwhile, the affected side of the thyroid gland and the greater horn of the hyoid bone should be removed to prepare for the tumor excision.

The main purpose of reconstruction of LF is to prevent aspiration after surgery. In patients with hypopharyngeal carcinoma, most of the ipsilateral supraglottic tissues have to be removed, which may lead to the laryngeal inlet being in an asymmetrical state. If the asymmetrical inlet is not corrected, the new laryngeal inlet cannot be sealed safely in the process of swallowing, and serious aspiration may occur, causing pulmonary infection and even threatening the survival of elderly patients. After resection of the lateral wall of the pyriform sinus or posterior wall carcinoma, most of the laryngeal tissues are preserved, except for the posterior part of the thyroid cartilage. Since the mucosa of the laryngeal cavity is intact, laryngeal reconstruction is unnecessary. However, for cases with tumors in the medial wall of the pyriform sinus or postcricoid area, the majority of the supraglottic tissues on the same side as the tumor are resected. In those cases, we took the following measures to prevent aspiration: (1) If the epiglottis was kept intact, the epiglottis was pulled downward or rotated outwards in order to suture it with the surgical margin of subglottic tissues and to re-shape the lateral wall of residual larynx. If the edge of the epiglottis was involved by tumor, the affected part of the epiglottis was removed and the remnant was pulled downward to reconstruct the lateral wall of the laryngeal vestibule and to cover the laryngeal inlet effectively [10, 11]; (2) The myofascial flap of sternohyoid muscle was sutured with the glottic or subglottic tissues to repair the defect of the lateral wall of the laryngeal cavity. The flap was inverted into the laryngeal cavity through the upper side of the thyroid cartilage to repair the defects of the larynx. The pedicels of epiglottis and myofascial flap of sternohyoid muscle were sutured to cover the laryngeal inlet and prevent aspiration. The sternohyoid muscle and the mucosa of epiglottic vallecula were sutured with the tongue base and elevated to the laryngeal inlet to protect the function of deglutition; (3) The aryepiglottic fold and postcricoid mucosa were pulled to the affected side and sutured with the posterior border of the vocal cord or ventricular cord, and this formed several bigger mucous folds in the posterior wall of the laryngeal inlet; (4) If most of the affected laryngeal tissue was removed and more than half of the epiglottis was removed, the remnant mucosa of larynx and hypopharynx was used to reconstruct the speech tube; (5) Once the defect of the laryngeal cavity was repaired, the larynx was pulled up and fixed to the hyoid, and the laryngeal inlet was kept closer to the tongue base as much as possible; and (6) If the epiglottis was resected completely, the tongue flap was sutured with the laryngeal inlet and the larynx was pulled up near the tongue base. The tongue flap covered the laryngeal inlet and the pyriform sinus was capacious to help the food pass the laryngeal inlet as quickly as possible to avoid aspiration.

The thorough resection of tumor, amount of remnant laryngeal tissue, and patient’s general condition are the key factors considered when we take LF preservation into account. LF preservation is not suitable for old or weak patients and those with poor cardiopulmonary function, even if there is enough remnant laryngeal tissue. The patients with at least one movable arytenoid cartilage and a normal contralateral pyriform sinus can have preservation of LF and reconstruction of the pharyngeal cavity. LF can be reconstructed when the posterior edge of the thyroid cartilage or the esophageal inlet is involved because of the adequate remnant of laryngeal framework. In most cases, the carcinoma in the pyriform sinus only involves the ipsilateral supraglottic structure of the larynx and is less aggressive to the contralateral laryngeal tissues. If the laryngeal framework stays intact and most of the laryngeal mucosa is conserved, tracheal decannulation is not difficult to construct and the patient’s speech will be close to normal. However, if the laryngeal cavity is involved by advanced cancer and the framework of the larynx is removed at a large scale or the new laryngeal inlets are blocked by repaired tissues, decannulation will be a problem and the voice will be hoarse and weak.

Because they have less laryngeal cavity infiltration, patients with carcinoma located in the posterior wall of the hypopharynx can have LF preservation, but LF preservation in the postcricoid area is troublesome. Our experience is that if the bilateral arytenoid cartilage is movable and the interarytenoid notch is not infiltrated, the carcinoma can be resected completely without total laryngectomy. The LF reconstruction materials include the upper part of the esophagus and remnant medial wall of the bilateral pyriform sinus. The mucosa of the esophagus and remnant pyriform sinus can be sutured together to reconstruct the continuity of the hypopharynx-esophagus. In the case that part of the stomach or colon with vessel rootstock was used to repair the hypopharynx or cervical esophagus, the laryngeal inlet is also elevated. Otherwise, excessive secretion from the stomach pull up or colon tissue would be regurgitated to the laryngeal cavity and cause severe aspiration. In this study, by postsurgical LF evaluation methods such as subjective sensation, voice quality, electronic laryngeal endoscopy, and X-ray barium meal examination, most of our patients (70.3 %) had overall LF recovery including respiration, voice and deglutition and approximately 30 % of them had partial LF recovery including voice and deglutition.

The most important aspect of hypopharyngeal function reconstruction is to create/maintain a spacious cavity in the hypopharynx so that food can pass more quickly and smoothly and aspiration will be less likely. Based on the experience from our clinic, the symmetry of the bilateral pyriform sinus should be emphasized and the restoration of the ipsilateral affected pyriform sinus must be taken into consideration during the operation. In our practice, we have found that if the mucosa is intact in the opposite pyriform sinus and postcricoid area, a defect at one side of the pyriform sinus will not influence swallowing function severely. It is important to deal appropriately with the laryngeal inlet rather than to rebuild the affected ipsilateral part of the pyriform sinus. For early stage tumors, after the tumor in the pyriform sinus or posterior wall is removed, the remnant mucosa can be sutured directly to close the hypopharyngeal cavity. However, for advanced-stage tumors, the removed tissues exceed the ipsilateral area in the pyriform sinus and extend to the posterior wall of the hypopharynx, postcricoid area, or cervical esophagus, and reconstruction of the hypopharynx should use the myocutaneous flap of the pectoral major muscle or part of the stomach pull up or colon with vessel rootstock. In order to relieve tension in the anastomotic inlet, the elevated stomach pull up or colon can be fixed with surrounding tissue at first and then anastomosed with the mucosa of the hypopharynx; then the anastomotic inlet can be made oblique and big enough to avoid food regurgitation and aspiration during deglutition. In cases with removal of one side of the lateral wall of the cervical esophagus and tumor involvement limited to within 3 cm below the inlet of the esophagus, a myocutaneous flap of pectoral major muscle or free jejunum flap is still suitable to repair the defect. However, the anastomotic orifice should be formed obliquely to prevent anastomosis from stenosis [12, 13]. However, if the cervical esophagus is involved more than 3 cm, the usage of a free jejunum flap is difficult because the esophagus incisal margin is below the superior aperture of the thorax. In that case, the stomach pull up or colon tissue with vessel rootstock is more appropriate in our experience [14]. The myocutaneous flap of pectoralis major muscle is sutured with the remnant of hypopharyngeal mucosa to repair the cavity of the hypopharynx. When most of the mucosa in the inlet of the cervical esophagus is resected, a stenosis is often formed in the stoma; therefore, the hypopharynx or the cervical esophagus is reconstructed with either a myocutaneous flap of the pectoralis major muscle, stomach pull up or colon tissue with vessel rootstock, or a free jejunum flap [15].

In summary, although the primary goal of hypopharyngeal carcinoma surgery is still thorough tumor resection, LF preservation does not have to come at the cost of a safe margin. Moreover, our results indicate that patients with LF preservation had significantly better survival than those without LF preservation. Therefore, the LF preservation of patients with hypopharyngeal carcinoma should be advocated whenever possible in order to improve the clinical outcomes of such patients [16, 17].

## Electronic supplementary material

Below is the link to the electronic supplementary material.

Supplementary material 1 (TIFF 332 kb)

## Abbreviations

LF: Laryngeal function
TNF-α: Tumor necrosis factor-alpha
SCCHN: Squamous cell carcinoma of the head and neck
HR: Hazard ratio
CI: Confidence interval
PCR: Polymerase chain reaction
SNPs: Single nucleotide polymorphisms
SCCNOP: Squamous cell carcinomas of the nonoropharynx
