# Supplementary figures and images for: Preservation of laryngeal function improves outcomes of patients with hypopharyngeal carcinoma
Source: Eur Arch Otorhinolaryngol. 2014 Jun 10;272(7):1785–91. doi: 10.1007/s00405-014-3115-2 (PMC4438222; doi:10.1007/s00405-014-3115-2)

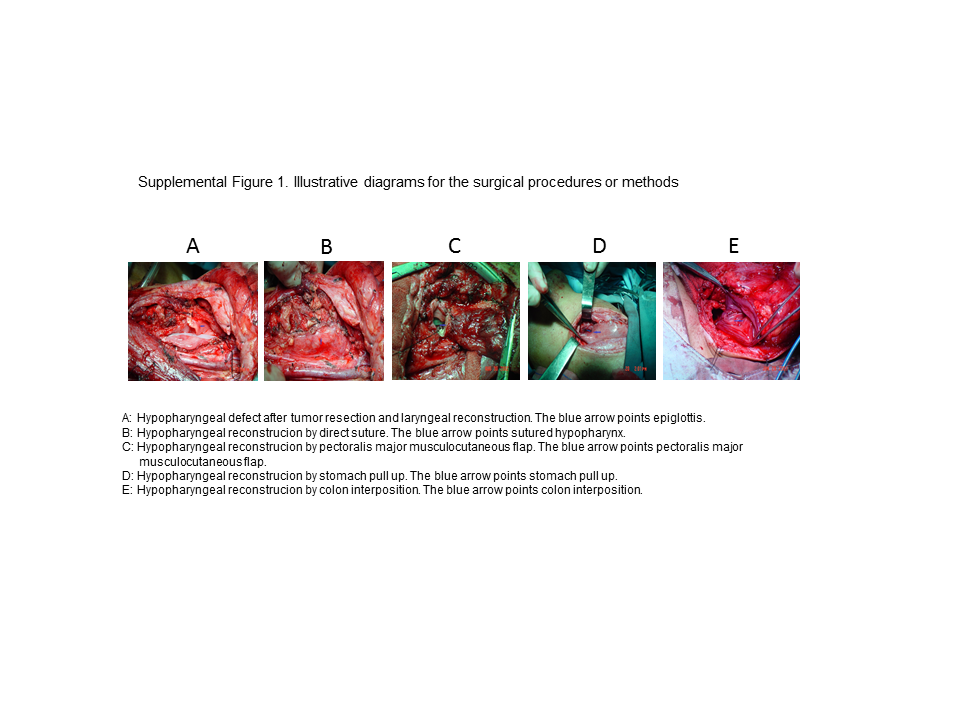

Supplement: Supplementary file 1 — Supplementary material 1 (TIFF 332 kb) [file 405_2014_3115_MOESM1_ESM.tif]
